# Unmet needs in ANCA-associated vasculitis: Physicians’ and patients’ perspectives

**DOI:** 10.3389/fimmu.2023.1112899

**Published:** 2023-02-23

**Authors:** Luca Quartuccio, Elena Treppo, Livio Urso, Giulia Del Frate, Federica Mescia, Federico Alberici, Augusto Vaglio, Giacomo Emmi

**Affiliations:** ^1^ Division of Rheumatology, Department of Medicine, University of Udine, Azienda Sanitaria Universitaria del Friuli Centrale, Udine, Italy; ^2^ Department of Experimental and Clinical Medicine, University of Florence, Florence, Italy; ^3^ Nephrology Unit, University of Brescia, Azienda Socio Sanitaria Territoriale Spedali Civili, Brescia, Italy; ^4^ Nephrology and Dialysis Unit, Meyer Children’s Hospital, Florence, Italy; ^5^ Department of Biomedical, Experimental and Clinical Sciences, University of Florence, Florence, Italy; ^6^ Centre for Inflammatory Diseases, Monash University Department of Medicine, Monash Medical Centre, Clayton, VIC, Australia

**Keywords:** ANCA, challenges, patient report outcome, vasculitis, biomarker, therapy

## Abstract

In recent years, clinical research has increased significantly and therapies for antineutrophil cytoplasmic antibody (ANCA)-associated vasculitis have improved. However, there are still unanswered questions and unmet needs about AAV patients. The purpose of this review is to examine the frontiers of research related to emerging biomarkers eventually predicting relapse, and new therapeutic approaches, not to mention new quality of life assessment tools. Identifying predictors of relapse may help optimize therapeutic strategies, minimize disease recurrence, and reduce treatment-related side effects. In addition, it is important to recognize that patients may suffer long-term consequences of the disease and its treatment, which, although life-saving, is often associated with significant side effects. Our goal, therefore, is to highlight what has been achieved, the pitfalls, and what still needs to be done, comparing the views of physicians and patients.

## 1 Introduction

Antineutrophil cytoplasm antibody (ANCA)-associated vasculitis (AAV) is a group of rare systemic disorders characterized by inflammation of small-sized blood vessels and comprises three entities: granulomatosis with polyangiitis (GPA), microscopic polyangiitis (MPA), and eosinophilic granulomatosis with polyangiitis (EGPA) (1). Further entity is represented by single organ disease, i.e. renal-limited vasculitis. Updated Classification Criteria for AAV (2–4) have been recently endorsed by American College of Rheumatology (ACR) and European Alliance of Associations for Rheumatology. Otherwise, no diagnostic criteria are available for AAV to date.

Despite increased clinical research and improved therapies over the last years, there are still unanswered questions and unmet needs of AAV patients. Herein, our purpose is to describe the frontiers of research concerning emerging biomarkers, novel therapeutic approaches, and the physicians’ perspectives, while also reporting new quality-of-life assessment tools, which emphasizes patients’ perspectives.

## 2 Antineutrophil cytoplasmic antibodies: clinical and therapeutic significance

The definition of AAV is itself closely linked to the presence of circulating autoantibodies, i.e. ANCA, which have proteinase 3 (PR3) and myeloperoxidase (MPO) as their main target antigens. Although the diagnostic value of ANCA is well established, the presence of patients with AAV without ANCA positivity, i.e. seronegative disease, represents a challenge for researchers and clinicians. It is well known that ANCA are found in approximately 70-80% of patients with GPA and MPA, with even smaller percentage in limited forms (e.g renal of ENT limited AAV) (5–7). On the other hand, EGPA is often ANCA-negative, and only 30% of EGPA patients shows ANCA, mainly with an MPO-ANCA specificity. A necessary consideration is that, in seronegative disease, anti-MPO and anti-PR3 may be present but remain below the detection limit of current enzyme immunoassays or the masking of the epitope may confuse their detection (8, 9). Moreover, current mainstream ELISAs for ANCA detect IgG, thus, patients who are negative for IgG ANCA, may have an IgA ANCA disease (10).

### 2.1 Beyond ANCA positivity: emerging biomarkers in the seronegative disease

Pentraxin 3 (PTX3), is an acute-phase reactant produced by innate immune cells in response to inflammatory stimuli, recently emerged as an interesting novel serological biomarker for various autoimmune diseases (11). PTX3 is a protein structurally similar to the short pentraxin C-reactive protein (CRP), and preformed PTX3 is natively stored in neutrophil granules, like PR3 and MPO, and can localize in neutrophil extracellular traps (NETs). Unlike CRP, which is mainly produced in the liver, PTX3 is widely produced by a variety of cells, including dendritic cells, macrophages, endothelial cells and renal epithelial cells. It is believed to reflect local tissue inflammatory activity more closely than CRP. Furthermore, it has been shown to influence the regulation of glycosylation-dependent inflammation, to dampen the recruitment of neutrophils (12) and to contribute to the activation and regulation of complement pathway (13). By indirect immunofluorescence, anti-PTX3 gave rise to a specific cytoplasmic fluorescence pattern distinct from the classical cytoplasmic, perinuclear or atypical pattern. A recent study showed that anti-PTX3 antibodies correlated with the Birmingham Vasculitis Activity Score (BVAS) at baseline, and plasma and urinary PTX3 levels are increased in active AAV disease, especially in patients with renal involvement (14). Anti-PTX3 was detected in approximately 30-40% of AAV patients and, interestingly, also those negative for MPO- and PR3-ANCA (15, 16). Therefore, anti-PTX3 autoantibodies might help the clinician especially in cases of seronegative AAV (16).

Previous studies already suggested the potential role of other antibodies, namely the lysosomal-associated membrane protein-2 (LAMP2) in ANCA-negative disease; in fact, anti-LAMP2 antibodies was found in pauci-immune focal necrotizing glomerulonephritis even in the absence of antibodies to MPO and PR3 (17). LAMP2 is a heavily glycosylated membrane protein and it is co-expressed on neutrophils with MPO and PR3 as a target of ANCA and is commonly included in the group of “minor” ANCAs.

### 2.2 How should we treat ANCA-negative AAV?

The absence of ANCA still represents a challenge for physicians, in fact, ANCA-negative patients were excluded from the majority of RCTs and the optimal therapeutic strategy mainly relies on case series (18). If it is true that seronegative patients have more limited disease and less frequently a kidney involvement (19), their prognosis is not necessarily good: while a Chinese multicentre retrospective study found a better overall survival in negative patients as compared to MPO- and PR3-ANCA patients (20), on the contrary, in the French Vasculitis Study Group Registry, ANCA-negative and ANCA-positive patients have similar relapse free survival and overall survival after statistical adjustments (19).

Thus far, according to available data, treatment strategy do not differ based on serological status: in a recent case series of 73 ANCA-negative pauci-immune necrotizing glomerulonephritis, patients were treated for remission induction mainly with high-dose corticosteroids and cyclophosphamide (CYC); rituximab (RTX) was used in 5 patients despite the absence of detected pathogenic autoantibodies, although vasculitis remission was obtained only in one patient (21). In previous case series involving ANCA-negative patients with severe disease, RTX was able to induce remission (22), highlighting a role for B-depleting therapy beyond autoantibodies suppression, including the B-mediated reduction of CD8+ T cell cytokine production (23). Finally, RTX is a viable alternative to methotrexate and mycophenolate in granulomatous/localized GPA, usually ANCA negative (24).

## 3 Challenges in management and disease monitoring

Based on accumulating evidences during decades (randomized clinical trials/observational studies), the current approach to patients with severe AAV (organ or life-threatening disease) consist of an induction treatment, aiming to halt tissue damage with high dosage of potentially cytotoxic drugs, followed by a maintenance treatment, aiming to stabilize remission with favourable safety profile drugs. A complete review of such therapeutic strategies and the trials which support them, has been recently reviewed elsewhere (25, 26). Although a certain international consensus exist regarding patient management (2021 American College of Rheumatology/Vasculitis Foundation Guideline (27) and 2016 European League Against Rheumatism/European Renal Association recommendations (28), several unanswered questions still persist.

The current induction therapy of severe AAV consists of GC combined with either RTX or CYC. CYC was the first drug shown to provide successful treatment and revolutionized the management of AAV (29), improving the prognosis the prognosis from a fatal disease to one that has >90% remission rate (30). The CYCLOPS trial (29) compared daily oral (DO) versus intravenously (IV) pulse CYC in patients with newly diagnosed severe renal GPA or MPA, showing a lower cumulative dose of CYC (8.2 g versus 15.9 g; p < 0,001) and a lower rate of leukopenia (hazard ratio [HR] 0.41) in the IV group. Differently, in the long-term follow-up, relapses were significantly lower in DO CYC than IV pulse CYC (HR 0.50), without any difference in mortality or renal function. Subsequently, two randomised trials, RAVE (31) and RITUXVAS (32), demonstrated that RTX was non-inferior to CYC in inducing remission in patients with both new and relapsed GPA and MPA. Even more in detail, RTX was more effective in relapsing disease (odds ratio [OR], 1.40) (31) and PR3-ANCA AAV compared with CYC (33). Thus, in 2011 RTX was approved by the Food and Drug Administration (FDA) in the treatment of patients with GPA and MPA. To date, despite the known potential risks associated with use of CYC (e.g. infertility, urotoxicity, haematological toxicity, infection), CYC still has a role in the management of patients with severe life-threatening disease, central nervous system (CNS) involvement and severe presentations of EGPA, including cardiac involvement.

Once remission is achieved, its maintenance is important to prevent relapse of the disease. The usefulness of ANCA for monitoring disease activity has also long been debated (34). Clinical studies showed a direct association between elevated PR3-ANCA levels during complete remission and increased risk of relapse, especially for renal or pulmonary disease (35), whereas others found a direct association between PR3-ANCA-positive patients and the likelihood of relapse compared with MPO-ANCA patients (36). However, relapse may occur without detection of circulating ANCA and ANCA positivity may persist even when the disease is in remission (35). Similarly, the presence of circulating CD19+B cells as a risk factor for disease relapse in AAV has long been debated due to conflicting data on this topic over the years (37). Despite these uncertainties, it has to be underlined that in the context of RTX treated patients, disease flares without at least one event between B-cells repopulation or rise of ANCA titre are unusual (38, 39) and the combined used of both biomarkers may prove useful in clinical practice.

The research for non-invasive biomarkers which can predict disease activity, prognosis and treatment options remains a much sought-after goal of many AAV studies. A number of serum and urinary biomarkers have been investigated as candidate tools for vasculitic activity (**Table 1**).

**Table 1:** Strengths and weaknesses of potential biomarkers (in alphabetical order).

Biomarker	Potential utility	Pitfalls
ANCA (*serum*)	Diagnostic value is well established	Discordance with disease activity: -Persistent positivity of ANCA in remission disease -Significance of reappearance of ANCA - Seronegative disease
Anti-LAMP2 Ab (*serum*)	Potential role in pathogenesis and association with disease activity	Limited role of “minor ANCA”
Anti-PLG Ab (*serum*)	Associated with higher degree of acute inflammatory renal lesion (potential biomarker of renal severity)	Associated with ANCA seropositivity; Unknown prognostic utility
Anti-PTX-3 Ab (*serum*)	Proposed role as a diagnostic biomarker in ANCA-negative AAV; Potential in distinguishing disease activity status	Lack of widespread routine tests (need for reference laboratory)
BAFF (*serum*)	Potential therapeutical role of anti-BAFF agents	Conflicting data between correlation of BAFF levels and disease activity in AAV
Bregs and naïve B-cells	Crucial in maintaining self-tolerance; Inverse correlation between CD5+ B-cells and disease activity; Lower relapse risk with repopulation of naïve CD19+CD27- B-cells	Role limited to patients treated with RTX; Need for reference laboratory and sensitive flow cytometry method
CECs	Correlation with disease activity	Lack of widespread routine tests
Complement fractions	Correlation with disease activity (Bb, C3a, C5a, and soluble C5b-9); Prognostic value in renal involvement (serum C3 levels); Therapeutic target (C5a receptor)	Not association between serological and pathohistological phenotypes and serum C3 levels
EMPs	Correlation with disease activity	Lack of widespread routine tests
EPCs	Inverse correlation between EPCs levels and relapse risk	Lack of widespread routine tests
sCD163 (*urine*)	High sensitivity and specificity for active renal vasculitis	Limited role in renal involvement disease; Does not distinguish infection from relapse; Not disease-specific (has also been studied in SLE)
S100A8/S100A9 (*serum*)	Correlation with disease activity	Reduction but not normalization into disease remission (subclinical inflammation)?; Not disease-specific
T-cells	Inverse correlation between CD25+ T-cells and disease activity; Correlation between Th17 cells and disease activity; Correlation between CD8+ T-cells levels and relapse risk	Further researches are needed
uMCP-1 (*urine*)	Proposed role in renal AAV disease activity; Increasing levels during renal flare of disease	Limited role in renal involvement disease

ANCA, anti-neutrophil cytoplasmic antibody; Ab, antibodies; BAFF, B-cell activating factor; Bregs, regulatory B-cells; CECs, circulating detached mature endothelial cells; EGPA, eosinophilic granulomatosis with polyangiitis; EMPs, endothelial microparticles; EPCs, endothelial progenitor cells; LAMP2, lysosome-associated membrane protein-2; uMCP-1, urinary monocytes chemoattractant protein-1; PLG, plasminogen; PTX-3, pentraxin-3; sCD163, soluble CD163; S100A8/S100A9, calprotectin.

### 3.1 How to predict relapses or sustained remission?

Emerging research on B-cell phenotype have shown promising results in predicting AAV flares (40). Regulatory B-cells (Bregs) are crucial in maintaining self-tolerance through IL-10 production, and a surrogate marker for Bregs in AAV has been found in circulating CD5+ B-cell. The repopulation of B-cells after RTX with a percentage of CD5+ B-cells lower than 30% had been correlated with shorter relapse-free survival period (40). Similarly, repopulation of naïve CD19+CD27- B-cells 6 months after the first RTX treatment has been shown to be protective towards the risk of relapse (41), and intriguingly this subset included CD19+CD24^high^CD38^high^CD27- Bregs. Moreover, high circulating CD27+CD38^high^ B-cells count was correlated to decreased relapse-free survival in GPA (42). Recently, in RTX treated patients, a higher proportion of autoreactive PR3+ plasmablasts at the time of B-cells recurrence has been associated to a higher relapse risk (43). Importantly, it has been shown that B-cell depletion after RTX is never absolute, depending on the sensitivity of the flow cytometry method. ANCA-specific memory B-cells and CD20- plasma cells remain detectable after RTX (44). All these findings are still incomplete and debated, and improvement in the analysis of B-cells subset may contribute to a better definition of their role as biomarkers. Of interest, increased B-cell activating factor (BAFF) levels have also been reported in AAV and it may potentially hinder RTX effect. Of note, a single nucleotide polymorphisms in the regulatory region of the BAFF gene, has been found associated to a higher risk of RTX failure at 6 months and a shorter time to RTX failure after a RTX based induction; of note the carriers of the unfavorable genotype were more likely to have detectable circulating B-cells 6 months after RTX as well as a lower IgM reduction (45). The use of RTX and anti-BAFF agents in sequence by abolished auto-reactive B-cells survival has therefore a strong rational (46), even if the clinical utility of this combination treatment remains unvalidated. Despite the suggestion, the clinical utility of this combination treatment remains unvalidated.

Furthermore, considering that the alternative complement pathway plays a crucial role in the pathogenesis of AAV, measurement of complement fractions may also serve as a biomarker. It has been demonstrated that urinary levels of Bb, C3a, C5a, and soluble C5b-9 are significantly higher in active disease (47) and low serum C3 levels at diagnosis are associated with worse patient and renal outcomes in AAV patients (48). Of interest, the demonstration that the lack of C5a receptor induced resistance to ANCA-induced disease (49), namely experimental anti-MPO vasculitis with the significant attenuation of the neutrophil glomerular influx and lower albuminuria, showing once again the close link between biomarker research and the advancement of therapies. In addition to the actors mentioned above, the pathogenesis of AAV is also related to T cells. In patients with AAV, an increase in T helper 17 (Th17) cells and a decrease in regulatory T cells (Treg) has been observed, as well as a tendency for a decrease in IL-2 and IL-4 and an increase in IL-6, IL-10, TNF-α, IFN-γ and IL-17A (50). An increase in circulating follicular helper (Tfh) T cells (CD4+CXCR5+CD25-CD127^interm-hi^), a decrease in follicular regulatory (Tfr) T cells (CD4+CXCR5+CD25+CD127^lo-interm^) and an elevated Tfh/Tfr ratio compared to healthy controls was also observed in AAV patients (51). To date, little is known about the role of Tfr cells in vasculitis.

### 3.2 The frontiers of B cell treatment: how will we treat refractory/relapsing patients?

B cell depletion is an efficacious and widely used strategy to treat AAV, however incomplete B cell depletion or immunogenicity (52) affect the success of RTX therapy. Alternative anti-CD20 monoclonal antibodies has been tested with promising preliminary results: ofatumumab (53) and obinutuzumab (54) demonstrated a good safety and efficacy profile in small case series.

Another mechanism for depleting B cells is to target the CD19, an antigen presented by autoantibody-secreting plasmablasts and plasma cells as well as early B-cell, differentiation stages not covered by anti-CD20 therapy (55). Clinical efficacy of the anti-CD19 monoclonal antibody obexelimab has not yet been demonstrated (56), but chimeric antigen receptor T (CAR-T) cells engineered to recognize CD19 (antiCD19-CAR-T cell therapy) seems feasible, tolerable and highly effective in active systemic lupus erythematosus (57).

As mentioned above, since proinflammatory B-cell could paradoxically arise after RTX therapy due to high BAFF levels, a combination of anti-CD20 and anti-BAFF is currently under investigation (COMBIVAS trial, NCT03967925).

Finally, alemtuzumab is a humanised monoclonal anti-CD52 antibody which allows deep lymphocyte depletion. In refractory/relapsing CYC- and/or RTX-experienced patients, alemtuzumab provided a complete (BVAS 0) or partial (BVAS < 50% of baseline with no severe items) response in 75% of patients, although some concerns due to safety profile (58).

### 3.3 Emerging biomarkers: what’s next

In the physicians’ perspectives, novel biomarkers are needed to better understand the pathophysiology of AAV and stratify the severity of disease. At present, many biomarkers have been studied, but they do not yet have a use in clinical practice.

Urinary soluble CD163 (sCD163) has been presented as the most promising biomarker of renal vasculitis (59). It is the enzymatically cleaved form of CD163, a glycosylated membrane protein exclusively expressed by monocyte-macrophage lineage. It is a soluble form of a high-affinity scavenger receptor for the haemoglobin-aptoglobin receptor complex and works as an innate sensor for bacteria. Patients with small vessel vasculitis (which includes AAV) have significantly higher urinary sCD163 levels than patients in remission, disease controls or healthy controls. Unfortunately, it is currently only used in clinical trials and there are no routine tests for it in clinical practice. Others potential biomarkers of renal involvement in AAV are monocytes chemoattractant protein-1 (MCP-1) and anti-tissue plasminogen (anti-PLG) autoantibody. MCP-1 is a chemokine deriving from renal cell in response to inflammatory stimuli. It is responsible of macrophages recruitment and fibrotic response in mesangial cells. On the one side urinary levels of MCP-1 (uMCP-1) have been shown to increase in AAV patients with active renal disease and decrease after therapy (60). In addition, uMCP-1 was correlated with BVAS. On the other hand, PLG is a key component of fibrinolytic system and its presence was correlated with fibrinoid necrosis, severe glomerular inflammation, and increased thrombotic events in AAV patients (61). Anti-PLG has been observed in up to 25% of patients with anti-PR3 and anti-MPO positivity. However, further studies are needed to investigate their prognostic role.

Increasing interest is being generated by heterodimer calprotectin (S100A8/S100A9), which is a toll-like receptor-4 ligand present in neutrophils and monocytes and is elevated in many inflammatory conditions (62). Phagocytes have been shown to release this complex after their interaction with activated inflamed endothelium. After secretion, the complex binds to activated endothelial cells *via* heparan sulfate proteoglycans and triggers proinflammatory effects, such as increased secretion of CXCL8, upregulation of ICAM-1, and further recruitment of leukocytes (particularly neutrophils). In addition, the interaction leads to impairment of the integrity of the endothelial monolayer and induction of both caspase-dependent and caspase-independent cell death mechanisms, resulting in both apoptosis and necrosis. It has also been demonstrated that NETs contain calprotectin. These properties make calprotectin a potentially important mediator of tissue damage in AAV, in which endothelial activation and vascular injury are prominent. In addition, research is being conducted on other biomarkers of endothelial injury in AAV, such as endothelial microparticles (EMPs) (63), circulating mature detached endothelial cells (CECs) (64), and von Willebrand factor (VWF). EMPs are complex vesicular structures released by activated or apoptotic endothelial cells, while CECs are necrotic and highly activated endothelial cells that detach from the vessel wall. Both were positively correlated with disease activity in AAV (63, 64). In the event of endothelial injury, bone marrow-derived endothelial progenitor cells (EPCs) proliferate to carry out endothelial repair. These events have been described as predictive of early relapse in adults with AAV, in whom lower number of circulating EPCs have been observed (65). Despite efforts to find out the most effective biomarkers, their role in clinical practice is still limited and has not yet yielded clinically relevant results. Given the technical and laboratory limitations, it may be expected that in the near future the integration of the above biomarkers will support therapeutic decisions.

## 4 Novel therapeutic approaches: glucocorticoid-sparing strategies

Since their introduction in 1950s, glucocorticoids (GC) are the cornerstone of autoimmune disease therapy, included vasculitis; however, a number of related side effects such as serious infections and cardiovascular events reduce life expectancy and quality of life, therefore GC-sparing strategies are strongly advisable. Analysing data from the PEXIVAS trial (66) and the LoVAS trial (67) which randomized patients to receive a standard-dose regimen versus a reduced-dose regimen of GC, the latter was associated with a reduced risk of death and serious infections while not increasing the End Stage Kidney Disease (ESKD). Data on serious adverse events are less consistent, since they did not significantly differ between groups in the PEXIVAS trial and was instead lower in the reduced-dose regimen in the LoVAS trial (68). Of note, a more pronounced steroid sparing effect may be achieved combining different immunosuppressive drugs such as CYC and RTX in glucocorticoid-free maintenance strategies (69).

### 4.1 Avacopan use in real life practice

Avacopan is a new oral C5a-receptor competitive inhibitor that recently demonstrated efficacy in remission induction of AAV patients. In the phase III ADVOCATE study, avacopan compared with GC was non-inferior in inducing remission at week 26 and superior in sustaining remission at week 52. Moreover, patients receiving avacopan had better improvement in eGFR at 26 and 52 weeks and a decreased rate of GC-related adverse effects (66).

In 2021, avacopan was first approved for the treatment of GPA and MPA in Japan and subsequently by the US FDA and by the European Medicines Agency (EMA). Despite a lack of post-marketing surveillance data, the first real-world clinical experiences with avacopan in patients with active AAV have been reported. Due to its compelling safety profile, avacopan was used for achieving and maintaining remission in difficult-to-treat patients, i.e refractory or relapsing disease despite high-dose GS therapy, contraindications for the use of GC (obesity and/or diabetes) and, limited upper respiratory PR3-AAV (70). In detail, in a case series (70) of 8 European GPA and MPA patients, all patients achieved clinical remission within 6 months and only one patient experienced a relapse with pulmonary involvement 6 months after avacopan start. The event coincided with a reduction of avacopan dosing to 20 mg twice a day due to transport restrictions during the second wave in the COVID-19 pandemic. Regarding to renal involvement which was present in 5 of 8 patients, estimated glomerular filtration rate (eGFR) slightly improved in 4 patients (range +5 to +9 ml/min) and decreased in none. Overall, the GC Toxicity Index (GTI) (71) improved in half of the patients.

In the era of replacing GC treatment, avacopan is an attractive option in challenging scenarios. Noteworthy, assessment of complement system activation might enable to identify patients that benefit from a complement-targeted therapy (72). However, a number of questions remain to be answered in future studies: is avacopan superior to GC in patients with advanced renal failure and more severe disease? What is the effect of avacopan in extrarenal manifestations? Should avacopan be used as a potential maintenance treatment? Could avacopan be combined with drugs other than RTX or CYC?

### 4.2 Management of elderly patients

AAV have a peak incidence at age 65 to 75 years. Since this clinical subset have higher comorbidities, serious adverse events and mortality rate compared to younger patients (73), a dose reduction of major immunosuppressant is often practised (74). However, older patients are often underrepresented in randomized clinical trials and an evidence-based approach is lacking.

The CORTAGE trial showed that using an induction regimen based on a lower cumulative dose of GC and CYC in patients 65 years and older reduces serious adverse events, not affecting the remission rate (75). In a multicentre cohort study involving 93 elderly people, RTX therapy was associated with achievement and maintenance of remission in the majority of patients aged 75 years and older with AAV. The incidence of serious infections and death was high when combined with high-dose GC regimens (induction therapy), but not in the maintenance therapy, suggesting a need for better prophylactic measures and probably less GC exposure (76). These issues need to be addressed in future studies.

### 4.3 Impact of ethnicity in AAV severity and treatment approach

Geographic variations have long been described in AAV, which may be a combination of genetic factors, and the effect of location in terms of latitude and UV radiation (77). Overall, a higher incidence of GPA is reported in north Europe and Australia, while a higher incidence of MPA is reported in Asia, especially in Japan. Thus, AAV has been most frequently studied in Caucasians and in high-income countries. May the ethnicity have a role in different response to treatments and/or severity of disease? Furthermore, the fact that access to care (not to mention access to specialized centers) is not equal in high- and in low/middle-income countries represent a further confounding factor in studies. In a recent study conducted in southern California (78), Hispanics had greater number of AAV relapses, a greater risk of developing acute respiratory failure (OR 1.33) and ESRD (OR 1.22) compared with Caucasians despite similar BVAS and VDI. Regarding renal involvement, no differences by race were found in treatment response, renal recurrence, ESRD or death between African-American patients with pauci-immune glomerulonephritis (GN) and Caucasians, although the former were found to be younger and more often MPO-ANCA positive (79). This data was confirmed by other single-center studies (80, 81), where no differences in disease manifestations, disease activity, and outcomes were observed between Black and White patients with AAV. In contrast, in a previous study (82) AAV-related renal disease seemed to be more severe in African Americans than in white Americans. In Latin America, extreme population heterogeneity has also been noted in AAV cohorts (83–85): in a Brazilian monocentric study (83), 75.7% of patients were White, 20.4% were Mulatto, 1.9% were Black, and 1.9% were Asian. The therapeutic approach did not differ from European guidelines (86), and the main predictors of mortality were serum creatinine level, C-reactive protein level, and VDI score (83). Survival was significantly lower in Brazilian patients with impaired renal function than in patients with normal renal function, and the main cause of death was infection (83). Comparing Asian and European studies, ESRD and renal involvement were lower in Korean and Indian patients with GPA than in Caucasians (87), probably due to the higher prevalence of limited/granulomatous GPA phenotype in those countries (88). There were no differences in the frequency of renal involvement in GPA between Japanese, Chinese and Western patients (87), although MPO-ANCA positivity was the predominant ANCA subtype in Chinese and Japanese patients, not only in MPA but also in the GPA phenotype (89, 90). MPO-ANCA positivity in Korean and Japanese MPA patients varied from 94 to 100% of patients and was remarkably higher than in Caucasians (87, 88). In contrast, Indian patients were more likely to have PR3-AAV similar to Caucasians, but were affected by GPA at an earlier age compared to Western populations. Overall, systemic GCs along with CYC were the mainstays for inducing remission in active GPA patients from Asia (91, 92), and mortality was higher in the first year of disease (87) due to disease itself or infections (91, 93). Very few studies have been conducted in Africa. In a retrospective study conducted in Senegal, more than two-thirds of the 27 cases of systemic vasculitis described were secondary forms (94). In another retrospective study (95) of 30 GPA patients conducted in Tunisia, ENT involvement was common during follow-up (83% of patients) but was rarely recognized at symptom onset (56% of patients). The renal involvement was frequent (56%) and significantly associated with poor prognosis (95). The rate of relapses was 36% and the overall 2-year survival was much lower (60%) than that reported for high-income countries. CYC was the treatment of choice as induction therapy and, notably, RTX has never been used despite the high frequency of ANCA positivity (90% of patients).

Although the impact of ethnic differences needs to be further clarified, the actual availability of more expensive therapies (i.e. avacopan) in low- and middle-income countries remains an additional confounding factor and limitation.

## 5 Quality of life (QoL): the point of view of AAV patients

Over the years, along with the introduction of novel drugs, AAV has evolved from an acute and severe disease to a chronic condition. Although the prognosis has greatly improved, patients suffer the long-term consequences of the disease and its treatment, which, although life-saving, is often associated with significant side effects. For this reason, the perspectives of physicians and patients do not always coincide.

### 5.1 Patient-reported outcome measures and clinical assessment tools

In a chronic disease management, the impact of symptoms (when disease is active), irreversible organ damage and treatment (including side effects of treatments) on patients’ health related quality of life (HRQoL) can be significant (96). This fact imposes the importance of patient-centered outcomes in routinary medical evaluation and in clinical trials (97).

Outcomes are traditionally measured through clinical evaluation and laboratory quantitative findings (e.g, creatinine to estimate the renal function, CRP or erythrocyte sedimentation rate [ESR] as measures of inflammation), together with clinical assessments such as BVAS (98) and the Vasculitis Damage Index (VDI) (99). However, the patient’s and the physician’s expectations and importance of outcome often differ (100, 101). It was shown that, despite being classified as in stable clinical remission, the patients’ opinion (in terms of Patients Global Assessment (PGA) assessing aspects of disease activity) differs in 40% of cases (102). Understanding the patients’ perspective allows physicians to understand patient goals and to better support them in their recovery (103). Patients’ interpretation of their HRQoL can be better assessed using Patient-Reported Outcome Measures (PROMs) (100). The inclusion of PROMs as outcome measures in AAV enables clinicians to provide a more patient-centered care, although, although patients are still often not taken into the right consideration in clinical studies. In a recent systematic review, it was found that only 41% of randomized controlled trial of AAV outcomes included PROMs) (97, 104).

AAV patient-reported outcome (AAV-PRO) questionnaire is an English language validated questionnaire, repeatable and self-administering in patients affected by AAV. It is composed of 29 items analyzing symptoms, difficulties of everyday life, social and emotional impact. These items are often unexplored by the clinician during the clinical evaluation. Hence, the AAV-PRO can guarantee for the patients that their perspective on the disease is represented in the study on AAV (100, 104). The effort to make AAV-PRO more useful and available in the clinical practice is ongoing: AAV-PRO has been translated in 14 languages and it has been validated in order to be incorporated into clinical trials (97, 105–107). Recently, a preliminary Italian work that translated and evaluated the AAV-PRO questionnaire showed that: a) AAV-PRO scores related with VDI value more than disease duration or activity; b) higher scores belonged to women in all domains, especially in ones concerning social and emotional impact; c) therapy with GC has impact on the physical function domain (105). These data are confirmed by a comparative Greek study (108) reporting that in AAV, QoL correlated more strongly with organ damage and less with disease activity, unlike in patients with rheumatoid arthritis, in whom QoL correlated with both disease activity and damage.

Given the above, it now seems reductive to define disease remission only as BVAS equal to 0. Is it time to rethink the concept of remission and low disease activity (LDA) in AAV, including steroid dose, ongoing treatment, and PRO in the evaluation? Defining novel therapeutic goals could guide in treatment (a kind of treat-to-target approach applied to certain rheumatic disorders) and in prediction of outcome in AAV. A persistent state of low disease activity (LDA) seem to be not a favorable target in AAV, in fact, in a recent study (109), the disease-related VDI score at 5-year follow-up was higher in the patients with prolonged LDA state and in those who never achieved LDA state compared to patients in prolonged clinical remission on treatment. These finding suggest that the accumulation of damage is more related to periods of poor control of disease activity than to treatment-related morbidity. Thus, the sustained achievement of any level of remission, regardless of the continuation of long-term immunosuppressive treatment, might potentially represent an optimal target to achieve better outcomes.

### 5.2 An underestimated symptom: focus on fatigue

A recent scoping review highlighted that patients with vasculitis are most affected physically by fatigue, psychologically by anxiety, socially by decreased social involvement and financially by functional decline and decreased employment (103). Currently, burdensome symptoms such as fatigue aren’t addressed in current management guidelines (28). In a recent study, fatigue has been stated as the most commonly reported symptom, with a prevalence up to 76% (110) and ranked among the top patient concerns; it is persistent, multifactorial (111–114), and not always can improve with treatment over time, affecting everyday life activities (115, 116). Luckily, there is an increased interest in defining fatigue and its origin for finding possible strategies to mitigate it (115, 117). Non-pharmacological interventions (maintenance of physical activity, healthy diet) should be considered to improve wellbeing, in association with pharmacological therapies (118). For example, a feasibility study of a behavioral-based physical activity intervention to support fatigue self-management in AAV patients has been conducted and accepted by patients (106).

### 5.3 Management of comorbidities and concept of frailty

Since AAV is a multisystemic disorder, many organs are involved. Thus, the management of comorbidities (both pre-existing and subsequent the diagnosis of AAV) is essential (119). As far as comorbidities are concerned, a significant increase in cardiovascular risk (in particular coronary artery disease and thromboembolic events), was evidenced during the active phase of the disease. AAV was also associated with bronchiectasis, thyroid diseases, osteoporosis, infections. Prospective studies on these comorbidities and their management (including a systematic screening and their treatment) are required in order to improve mortality, QoL and outcomes (116).

All the aspects listed above converge in the concept of frailty, syndrome characterized by a decreased physiological reserve and increased vulnerability to stressors, associated with disability and early mortality; it is not only a consequence of aging, but it is an aberrant process because of deficits in multiple systems. Frailty is combined with specific diseases and chronic conditions not necessarily associated with age, such as rheumatic diseases (120). In AAV both advanced age, inflammation and therapy contribute to frailty. As far as therapy is concerned, GC on one hand reduce chronic inflammation and allow good outcomes and survival, on the other can lead to sarcopenia, bone loss, higher risk of infection and thus frailty development (121). Although the prevalence of frailty in patients with AAV is unexplored, it was reported with the help of the VascStrong study that the majority of patients with vasculitis were rated as frail or pre-frail according to FRAIL scale (regarding AAV patients, up to 52% were identified as pre-frail and 20% as frail), with prevalence of female patients, obese and frequent use of GC therapy (122). A population of AAV patients was investigated by McGovern et al., highlighting that baseline frailty score was independently associated with mortality and for each additional point on the frailty score, the risk of death doubled; patients with lower and higher baseline score had no differences in time to remission or time to relapse, but in the frailer group a greater proportion of patients had adverse events, longer hospitalization time and higher mortality (123). Future investigations are needed to identify factors associated with frailty and to allow earlier identification and prevention.

## 6 Healthcare costs in AAV

AAVs are rare diseases, but imply very high healthcare costs. Both an Italian (124) and a German study (125) investigated the AAV burden of disease: AAV patients suffer from a high burden of morbidity, including multiple disease manifestations, relapses, and severe complications due to AAV treatment. Hospitalization was required most frequently because of the disease itself or superimposed infections, such as the development of end stage renal disease and this aspect was significantly related with the positivity of ANCA antibodies (124). In addition, the impact of novel, very expensive therapies (i.e. avacopan) will soon have to be assessed. In this context, there is a need to long-term studies to understand the course of disease and the safety of current therapies (27); this is important also in view of minimizing direct and indirect costs of AAV, and, impact on caregivers, considering that a quarter of them have reported some loss of income due to caregiving for systemic vasculitis (126).

### 6.1 Evaluation of work impairment in AAV patients

Work disability and productivity are increasingly recognized as important outcomes of interest in chronic disease because of the impact they have on individuals, families and society; work is an important part of life and contributes positively to a sense of wellbeing. With a move towards holistic care, there is increased emphasis on treating all domains of health (96). Although AAV most commonly occurs in older people (peak onset 65-74 years), many patients first develop vasculitis during their most production working years (118). There have been few studies looking at the impact of AAV on work disability (127–129). A recent Australian paper reported that about 25% of patients left paid employment due to the illness; most patients who were able to continue working reported that the disease had negatively affected their ability to work, and almost 50% of patients had their financial status impacted. Work impairment was associated with obesity, lower education level and fatigue. Nevertheless, the study doesn’t include a matched control group as a comparator, limit that should be overcome by future investigations (118, 127).

### 6.2 Upcoming technology in healthcare

Looking to future development, the integration of the technology within the healthcare is becoming more and more significant. Digital health (smartphone apps, sensors and wearable devices, video, social media platforms or messenger platforms) can overcome limitations of distance, location and time between physicians and patients. The opportunity to collect data electronically makes data acquisition faster and thus allows to a careful disease monitoring, intercepting the moment when there is any anomaly or deviation (for any reason linked to exacerbations/remissions of the disease, ongoing therapy or intercurrent events), helping to prioritize patients who may need further attention (130–132). In addition, there is a need for patient education, awareness programs, and support groups, in order to increase patient support across all health domains (103, 106).

## 7 Conclusion

**Figure 1:**
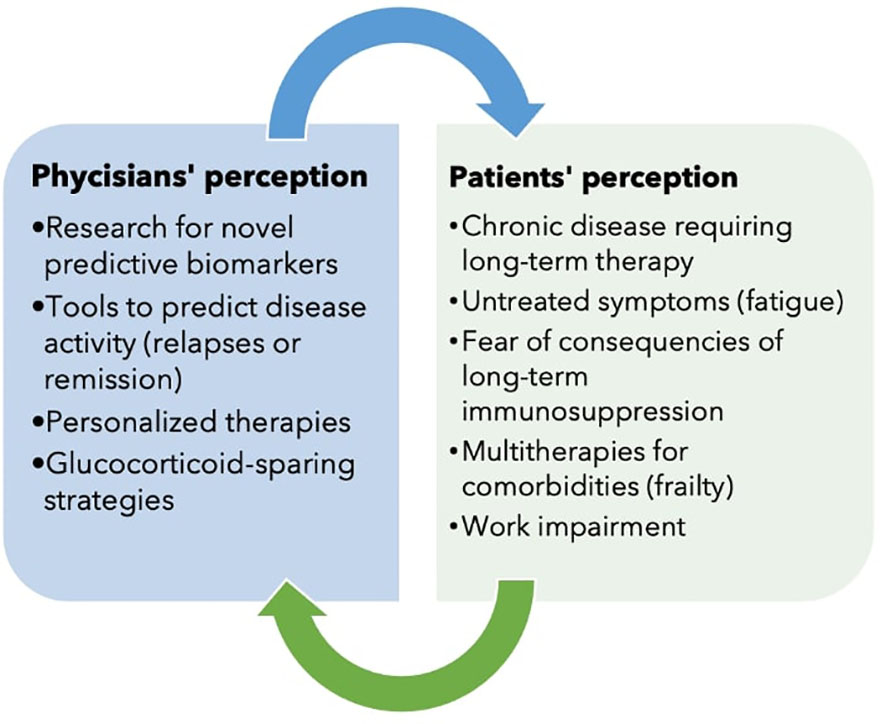
The physicians’ and patients’ perceptions compared: What matters most?

AAV is a group of rare multisystem diseases with a broad clinical spectrum that require a long-term immunosuppressive therapy. Over the years, AAV has evolved from an acute and severe disease to a chronic disease, changing the perspectives of physicians and patients in the management of the disease (**Figure 1**). We have attempted here to illustrate the many goals that have been achieved and those that remain to be achieved. Research is aimed at new biomarkers and therapies, but must not forget to consider the needs of patients as well. There are still many open questions and the research agenda is still long.

## Author contributions

LQ and GE conceived the study. The analysis was performed by LQ, GE, ET, GF, and LU. The first draft of the manuscript was written by LQ, GE, ET, GF, LU, and all authors commented on previous versions of the manuscript. All authors contributed to the article and approved the submitted version.
